# Circular RNA hsa_circ_0075542 acts as a sponge for microRNA-1197 to suppress malignant characteristics and promote apoptosis in prostate cancer cells

**DOI:** 10.1080/21655979.2021.1967064

**Published:** 2021-09-13

**Authors:** Yuefu Han, Xingqiao Wen, Xiaojuan Li, Dong Chen, Lian Peng, Bin Lai, Hongcai Huang

**Affiliations:** a Department of Urology, Zhujiang Hospital, Southern Medical University, Guangzhou, China; b Department of Urology, Yuebei People Hospital Affiliated to Medical College of Shantou University, Shaoguan, China; c Department of Urology, Third Hospital of Sun Yat-sen University, Guangzhou, China; d Department of Health Care, Shenzhen Hospital, Southern Medical University, Shenzhen, China

**Keywords:** Prostate cancer, tumor suppressor, competitive endogenous rna, miR-1197, hoxc11

## Abstract

Numerous differentially expressed circular RNAs (circRNAs) have been identified; however, their roles have not been fully elucidated. Since dysregulated circRNAs may have clinical applications, it is vital to study their expression characteristics, function, and mechanism in prostate cancer cells. The role, regulatory mechanism, and expression of hsa_circ_0075542 were analyzed using quantitative reverse transcription polymerase chain reaction. The results indicated that the expression of hsa_circ_0075542 was downregulated in prostate tumor tissues. The functions of prostate cancer cell lines LNCaP and PC3 cells were assessed using cell counting kit-8 and transwell assays and flow cytometry analysis. The results of the functional experiments showed that overexpression of hsa_circ_0075542 suppressed cell proliferation, reduced migration and invasiveness capabilities, and promoted apoptosis. Moreover, hsa_circ_0075542 targeted the microRNA-1197 (miR-1197) homeobox C11 (HOXC11) axis by sponging miR-1197. Overexpression of miR-1197 played a tumor-promoting role. Overexpression of hsa_circ_0075542 alleviated the tumor-promoting effect of miR-1197 overexpression In conclusion, hsa_circ_0075542 suppressed malignant characteristics and promoted apoptosis in LNCaP and PC3 cells by acting as a competing endogenous RNA of miR-1197. The hsa_circ_0075542/miR-1197 axis might play a role via HOXC11.

## 1. Introduction

Prostate cancer is an epithelial malignancy with the highest prevalence in males and ranks second and third in terms of morality in the United States and the United Kingdom, respectively [1–3]. An aging population, rapid economic development, and drastic lifestyle changes have led to an upward trend in the incidence of prostate cancer [4,5]. The early symptoms of prostate cancer are imperceptible, as they become evident only in the middle to late stages of the disease. Hence, it is vital to study the pathogenesis and mechanism of cancer development and progression to facilitate early diagnosis and ensure targeted therapy for patients with prostate cancer.

Circular RNAs (circRNAs) are abundantly present in the cytoplasm of eukaryotic cells. They are more stable than linear RNAs because of their covalently closed-loop structure [6]. The primary regulatory mechanism of circRNAs involves competitive endogenous RNA that regulates alternative splicing, translation, and expression of parental genes and interacts with RNA-binding proteins [7]. circRNAs can function as microRNA (miRNA) sponges and relieve the suppression of miRNAs on target genes [8,9]. circRNAs play an essential role in regulating the occurrence of various diseases, including cancers [6,10]. Many circRNAs, such as circFoxo3, circ_0044516, and circDDX17, are involved in disease progression and chemoresistance in prostate cancer [11–13]. Numerous differentially expressed circRNAs have been identified; however, their roles have not been fully elucidated. Therefore, studying the expression characteristics, function, and mechanism of dysregulated circRNAs in prostate cancer is crucial, owing to the potential clinical applications.

The expression profile and function of hsa_circ_0075542, identified as a downregulated circRNA in a previous study [14], was elucidated. Further, the regulatory mechanism of hsa_circ_0075542 based on the competitive endogenous RNA mechanism was identified. The results showed that overexpression of hsa_circ_0075542 suppressed cell proliferation, migration, and invasion and promoted apoptosis in LNCaP and PC3 cells by acting as a competitive endogenous RNA of microRNA-1197 (miR-1197). There is a need to further clarify the molecular mechanism of prostate cancer and provide a new potential target for targeted therapy.

## 2. Material and methods

### 2.1 Clinical tissue information

Prostate cancer and adjacent tissues (n = 30) were collected in accordance with the Helsinki Declaration of 1975, revised in 2013. All participants signed an informed consent form. All protocols were approved by the Ethics Committee of Zhujiang Hospital, Southern Medical University.

### 2.2 Quantitative reverse transcription polymerase chain reaction (qRT-PCR)

Total RNA was isolated from frozen clinical tissues and cell samples using TRIzol reagent (Invitrogen, Carlsbad, CA, USA). Reverse transcription of RNA to acquire cDNA was performed using a ImProm-IITM Reverse Transcription System (Promega, Madison, WI, USA). A reverse transcription primer was used for circRNA detection. PCR was performed with a SYBR GREEN qPCR super mix (Invitrogen); a 20 µL PCR mixture was prepared. The internal reference gene was beta-actin. The relative expression of hsa_circ_0075541 and hsa_circ_0075542 was calculated using the 2^−ΔΔCt^ method [15]. The forward and reverse PCR primers (5`–3`) used for hsa_circ_0075541, hsa_circ_0075542, and beta-actin were CTTTGTCACAGCTCGCATCA and CCAGATACACAGCATCATCT, ACCAAGGATTCAGTGTGGTG and GAGCTCCGGTATTCCAATAG, and GCATGGGTCAGAAGGATTCCT and TCGTCCCAGTTGGTGACGAT, respectively. The amplification products of hsa_circ_0075541 and hsa_circ_0075542 were sequenced using the Sanger method of DNA sequencing to confirm the occurrence of splice junctions.

### 2.3 Cell culture

Human prostate cancer cell lines, LNCaP and PC3, were purchased from the Cell Bank of the Chinese Academy of Sciences (Shanghai, China). All cells were cultured based on the instructions provided by the Cell Bank.

### 2.4 Recombinant plasmid construction and miRNA mimics

To simulate the overexpression of the hsa_circ_0075542 recombinant plasmid, the linear sequence of hsa_circ_0075542 (chr6:6,222,265–6,225,093) was cloned into the pLC5-ciR vector, a circRNA overexpression vector purchased from Guangzhou Geneseed Biotech Co. (China), using the following primers (5` – 3`): TTGACATTAATATTTCTTCTTTCGAATTCGTGAATGCCAAAGATGACGAAGGT (forward) and AGTATGGAGTTGTTAGCTAGGATCCCACACTGAATCCTTGGTGAGTTTGGAAT (reverse). The construction was named ov-circ_0075542. Negative control miRNA (miR-NC), miR-1197 mimic, miR-338-3p mimic, miR-548p mimic, miR-548 c-3p mimic, miR-545 mimic, and miR-634 mimic were purchased from Guangzhou RiboBio Co., Ltd. (China).

### 2.5 Cell transfection and cell groups

LNCaP and PC3 cells were transiently transfected with ov-circ_0075542 or the empty vector pLC5-ciR and were allocated to the ov-circ_0075542 group or empty vector group, respectively. LNCaP and PC3 cells were transiently transfected with miR-NC, miR-1197 mimic, miR-1197 mimic plus empty vector, or miR-1197 mimic plus ov-circ_0075542 and were named miR-NC group, miR-1197 group, miR-1197 + empty vector group, and miR-1197 + ov-circ_0075542 group, respectively. PC3 cells were transfected with miR-NC, miR-1197 mimic, miR-338-3p mimic, miR-548p mimic, miR-548 c-3p mimic, miR-545 mimic, or miR-634 mimic and were named miR-NC group, miR-1197 group, miR-338-3p group, miR-548p group, miR-548 c-3p group, miR-545 group, or miR-634 group, respectively. The transfection reagent used was Lipofectamine™ 2000 (Invitrogen).

### 2.6 Cell proliferation assay

Cells were harvested after transfection for 24 h and seeded in 96-well culture plates. After culture for 0, 1, 2, 3, and 4 days, cell proliferation assays were performed using a cell counting kit-8 (Dojindo, Japan), and the optical density at 450 nm (OD_450 nm_) of each group of cells was measured [16]. The following formula was used to calculate the cell proliferation rate: cell proliferation rate = (OD_450 nm_ value of 0, 1, 2, 3, or 4 days/OD_450 nm_ value of 0 days) × 100% (same group).

### 2.7 Flow cytometry analysis for apoptosis

Cells were harvested after transfection for 48 h and apoptotic cells were analyzed using an annexin V-FITC-propidium iodide apoptosis detection kit (Nanjing, China) on a BD FACSCalibur (BD Biosciences, San Jose, CA, USA) [16].

### 2.8 Transwell assay

A transwell assay was performed to assess the cell migration and invasion capabilities according to the methodology described in a previous study [17]. For the transwell migration assay, cells (1 × 10^5^) were harvested after transfection for 24 h and suspended in a serum-free medium. Approximately 1 × 10^5^ cells were seeded in the upper chamber of 24–well transwell chambers (BD Biosciences). Ham’s F-12 K medium or RPMI 1640 medium containing 20% fetal calf serum was added to the lower chamber to assay PC3 and LNCaP cells, respectively. After incubation in a humidified incubator with 5% CO_2_ at 37°C for 48 h, the non-migrated cells on the upper surface of the filters were wiped, and the upper chamber was fixed with 4% paraformaldehyde for 20 min. After washing once with phosphate-buffered saline solution, the migrated cells were stained with crystal violet for 10 min and then washed again with phosphate-buffered saline solution. Finally, the migrated cells were observed under a microscope. For the transwell invasion assay, the upper chamber of the transwell chambers was pre-coated with Matrigel (BD Biosciences) before cell seeding, and the protocol was the same as that used for the transwell migration assay. Five fields were randomly selected for imaging, and the migrated or invasion cell numbers in each field were counted. The average cell number was used to assess the migration or invasion capability of each group.

### 2.9 miRNA response element (MRE) prediction and luciferase reporter assay

The MRE of the hsa_circ_0075542 sequence was determined using Circular RNA Interactome online software (https://circinteractome.irp.nia.nih.gov/). The linear sequence of wild-type hsa_circ_0075542 was cloned into the miRNA target expression vector GP-mirGLO (Promega) to construct a luciferase reporter [18], named wild-type mirGLO-circ_0075542. miR-1197 MRE on wild-type mirGLO-circ_0075542 was mutated and this mutant luciferase reporter was named mutant mirGLO-circ_0075542. Wild-type mirGLO-circ_0075542 plus miR-NC, wild-type mirGLO-circ_0075542 plus miR-1197, mutant mirGLO-circ_0075542 plus miR-NC, or mutant mirGLO-circ_0075542 plus miR-1197 were transfected into 293 T cells. Values of *Renilla* and firefly luciferase activity were measured after transfection for 48 h using the dual-luciferase reporter assay system (Promega).

### 2.10 Biotinylated miRNA pull-down assay

Biotinylated miRNA pull-down assay was performed according to a previously described method [19]. Briefly, approximately 1.5 × 10^7^ LNCaP or PC3 cells, cultured in 60 mm tissue culture dishes, were transfected with 50 µM biotinylated miR-1197 mimics (biotin-miR-1197) or miR-NC (biotin-miR-NC). After transfection for 24 h, streptavidin magnetic bead preparation, cell lysis, pull-down incubation, bead washing, RNA precipitation, and RNA cleanup were performed as previously described [20]. The hsa_circ_0075542 level in the pulled-down RNA was determined with qRT-PCR, as described in sub-section 2.2. The amplification products were sequenced as described previously.

### 2.11 Protein extraction and quantification and western blot analysis

Total protein extraction, protein quantification, and western blot were performed according to the method described by Tan et al. [21]. The integral optical density of the gray scan of the western blot was measured using Image Pro-Plus 6.0 software (Media Cybernetics, Rockville, MD, USA). The relative expression of the target protein was calculated according to the method described by Tan et al. [21].

### 2.12 RNA fluorescence in situ hybridization

Fluorescence in situ hybridization of hsa_circ_0075542 and miR-1197 in PC3 cells was performed according to the method described by Tripathi et al. [22]. The hsa_circ_0075542 and miR-1197 probes were purchased from QIAGEN (Shanghai, China). The hsa_circ_0075542 and miR-1197 probes were labeled with TYE665 (red signal) and FAM (green signal), respectively, and the nucleus was stained with 4′,6-diamidino-2′-phenylindole (DAPI)blue signal).

### 2.13 Statistical analysis

Data were analyzed using GraphPad Prism version 7.0 (GraphPad Software, San Diego, CA, USA). The biotinylated miRNA pull-down assay results were analyzed using one-way analysis of variance, and the other results were analyzed using an unpaired *t*-test. A P value less than 0.05 was considered significant.

## 3. Results

Xia et al. [14] reported that expression of hsa_circ_0075541 and hsa_circ_0075542 was downregulated in four prostate cancer tissues. The present study showed that the expression of hsa_circ_0075542 was downregulated more than that of hsa_circ_0075541. Subsequently, the role and regulatory mechanism of hsa_circ_0075542 were explored *in vitro*. Our results showed that overexpression of hsa_circ_0075542 suppressed cell proliferation, migration, and invasion and promoted apoptosis in LNCaP and PC3 cells. Therefore, hsa_circ_0075542 might act as a competitive endogenous RNA of miR-1197.

### 3.1 Hsa_circ_0075542 level was downregulated

**Figure 1.**
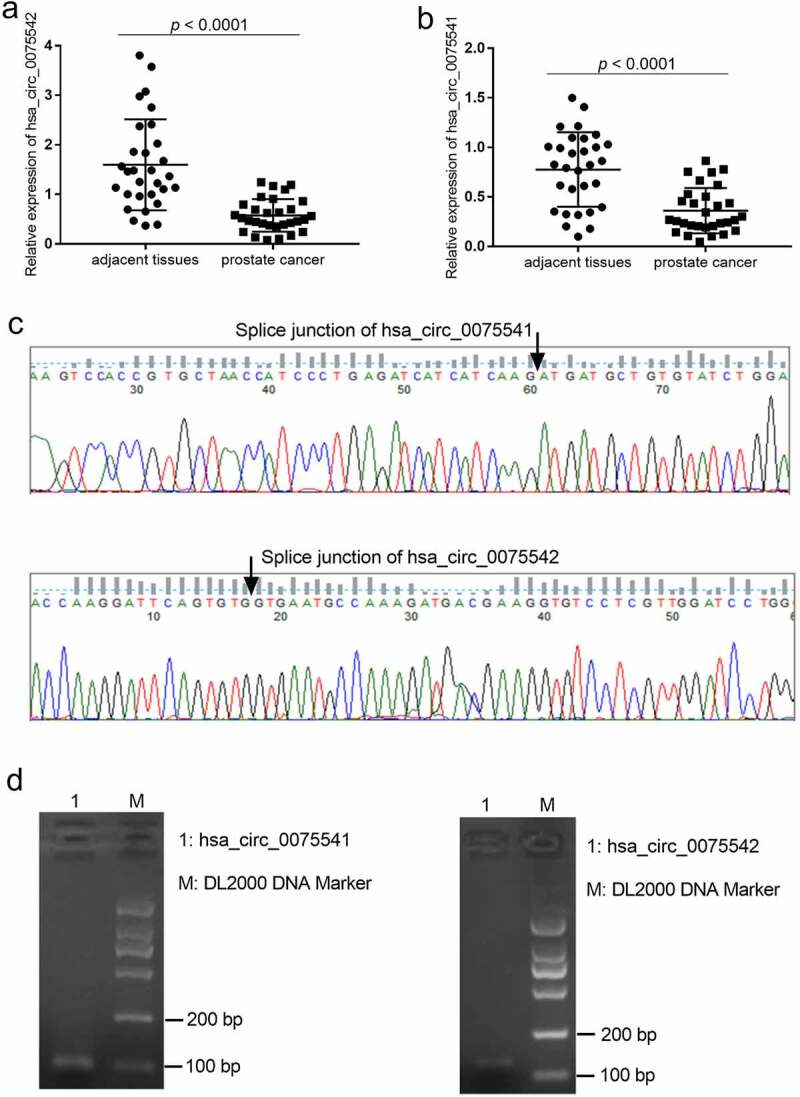
Expression of hsa_circ_0075541 and hsa_circ_0075542 in prostate cancer tissues. (a and b): hsa_circ_0075542 and hsa_circ_0075541 expression levels in prostate cancer and adjacent tissues, respectively; n = 30. (c): Sequence and (d): gel electrophoresis image of the quantitative reverse transcription polymerase chain reaction amplification products of hsa_circ_0075541 and hsa_circ_0075542

The expression of hsa_circ_0075541 decreased by approximately 50% (0.36 ± 0.23 vs. 0.78 ± 0.38) and that of hsa_circ_0075542 decreased by approximately 75% (0.58 ± 0.33 vs. 1.60 ± 0.92) in prostate cancer tissues when compared with those in adjacent tissues, respectively (Figure 1(a-b)). The splice junction of hsa_circ_0075541 and hsa_circ_0075542 was verified by the sequencing results of the qRT-PCR amplification products (Figure 1(c)). The length of the qRT-PCR amplification products was as expected (Figure 1(d)). The expression of hsa_circ_0075542 was downregulated decreased more than that of hsa_circ_0075541. Therefore, the overexpression of hsa_circ_0075542 in LNCaP and PC3 cells was subsequently studied.

### 3.2 Overexpression of hsa_circ_0075542 suppressed malignant characteristics and promoted apoptosis

**Figure 2.**
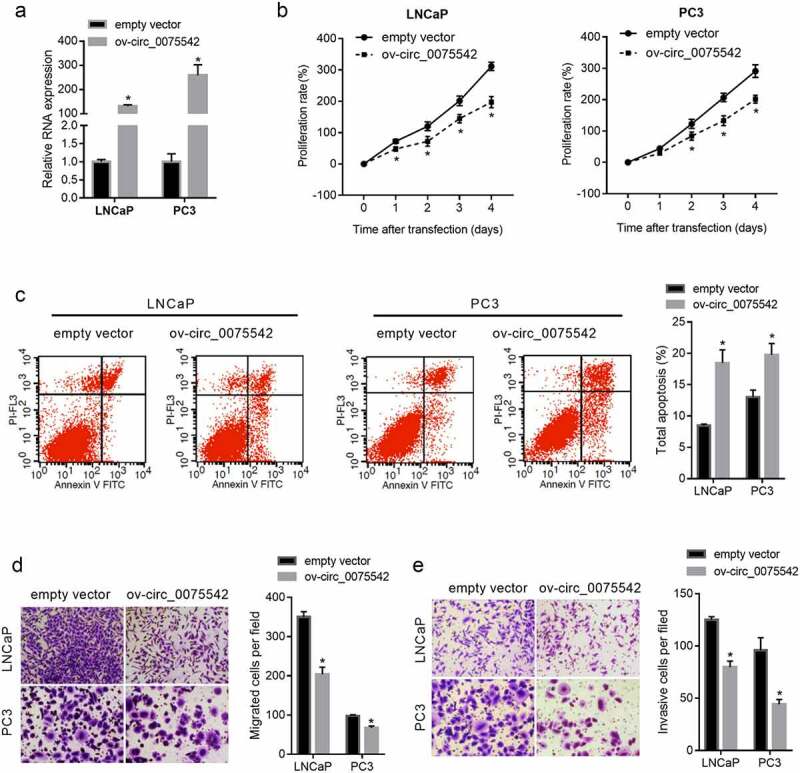
hsa_circ_0075542 plays a tumor-suppressive role in LNCaP and PC3 cells. LNCaP and PC3 cells were transfected with overexpression of hsa_circ_0075542 vector (ov-circ_0075542) or empty vector pLC5-ciR (empty vector). (A): hsa_circ_0075542 level in the above-mentioned transfected cells was determined using quantitative reverse transcription PCR. B-E: The effect of this transfection on cell proliferation (B) and apoptosis (C) was assessed using cell counting kit-8 and flow cytometry assays. The migration (D) and invasion (E) capabilities were evaluated using transwell assays. *P < 0.05, the ov-circ_0075542 group vs. the empty vector group

The hsa_circ_0075542 level increased 131-fold in LNCaP cells and 259-fold in PC3 cells (Figure 2(a)). The proliferation rate of cells transfected with ov-circ_0075542 was lower than that transfected with the empty vector (Figure 2(b)). The percentage of apoptotic cells was lower in the ov-circ_0075542 transfected cells than in the cells transfected with the empty vector (Figure 2(c)). Moreover, the migrated and invasive cell numbers were lower in the ov-circ_0075542 transfected cells than in the cells transfected with the empty vector (Figure 2(d-e)). Therefore, overexpression of hsa_circ_0075542 suppressed malignant characteristics and promoted apoptosis in prostate cancer cells.

### 3.3 Hsa_circ_0075542 targets the miR-1197/ homeobox C11 (HOXC11) axis

**Figure 3.**
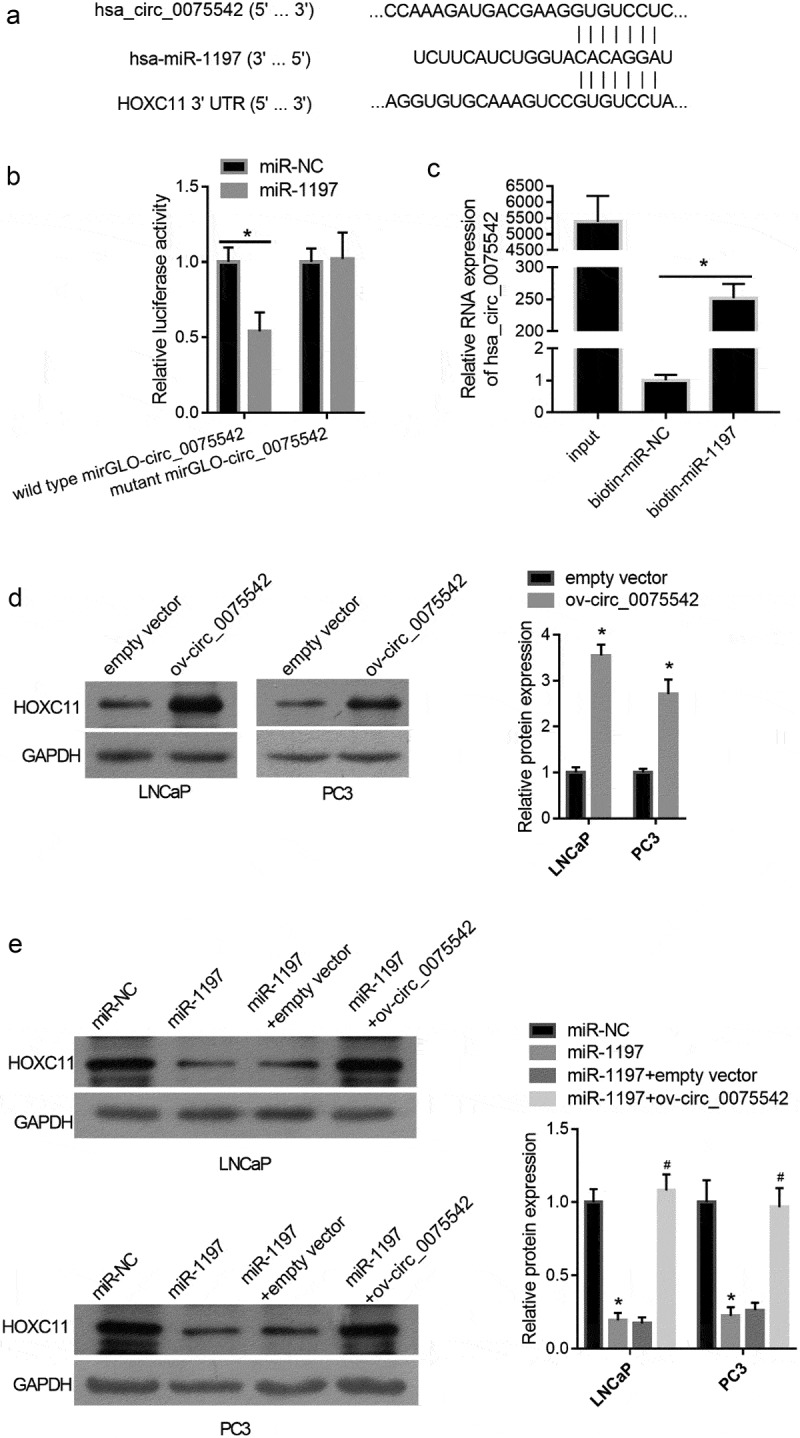
hsa_circ_0075542 targets the miR-1197/HOXC11 axis. (A): miR-1197 binding sites on hsa_circ_0075542 and 3′-noncoding region of HOXC11 mRNA. (B): Effect of miR-1197 mimic or miR-NC on the luciferase activity of recombinant luciferase reporter. To construct a luciferase reporter, the wild-type linear sequence of hsa_circ_0075542 was cloned into the miRNA target expression vector GP-mirGLO, named wild-type mirGLO-circ_0075542. miR-1197 binding site on wild-type mirGLO-circ_0075542 was mutated and the mutant plasmid was named mutant mirGLO-circ_0075542. miR-NC or miR-1197 was co-transfected with each recombinant luciferase reporter plasmid, and the luciferase activity was measured. (C): Relative expression of hsa_circ_0075542 in pulled-down RNA using biotinylated miR-NC or biotinylated miR-1197 as a probe. The ‘input’ group is the isolated RNA from the partial lysate before the cell pull-down assay. (D): HOXC11 protein level in cells transfected with overexpression of hsa_circ_0075542 vector (ov-circ_0075542) or empty vector pLC5-ciR (empty vector). (E): HOXC11 protein abundance in LNCaP and PC3 cells transfected with miR-NC, miR-1197 mimic, miR-1197 mimic plus empty vector, or miR-1197 mimic plus ov-circ_0075542. *P < 0.05

The MRE on the hsa_circ_0075542 sequence based on the competitive endogenous RNA mechanism was calculated. A context score percentile of 90 or greater in the Circular RNA Interactome online software was used as the cutoff value for MRE prediction. Six miRNAs, including miR-1197, miR-338-3p, miR-548p, miR-548 c-3p, miR-545, and miR-634, were found. Among these miRNAs, only miR-1197 promoted cell proliferation of PC3 cells (Fig. S1), suggesting that miR-1197 plays a tumor-promoting role, whereas the others play a tumor-suppressive role. Therefore, hsa_circ_0075542 might play its tumor-suppressive role by sponging miR-1197 based on the competitive endogenous RNA mechanism. The results of the luciferase reporter assay showed that miR-1197 mimic co-transfection had a lower relative luciferase activity of wild-type mirGLO-circ_0075542 than the miR-NC co-transfection, and the co-transfection of miR-1197 mimic and miR-NC had no effect on the mutant mirGLO-circ_0075542 (Figure 3(b)). The results of the pull-down assay using biotinylated miR-1197 showed that the expression of hsa_circ_0075542 was higher in RNA pulled-down using biotinylated miR-1197 than that pulled-down using biotinylated miR-NC (Figure 3(c)). Fluorescence in situ hybridization showed that hsa_circ_0075542 and miR-1197 were primarily co-localized in the cytoplasm (Fig. S2). These results verified the binding of miR-1197 on hsa_circ_0075542.

The effect of miR-1197 and overexpression of hsa_circ_0075542 on HOXC11 protein abundance were analyzed [23]. HOXC11 levels were upregulated by overexpression of hsa_circ_0075542 (Figure 3(d)), whereas they were downregulated by overexpression of miR-1197 (Figure 3(e)). Furthermore, HOXC11 levels were higher after miR-1197 + ov-circ_0075542 transfection than miR-1197 + empty vector transfection (Figure 3(e)), indicating that overexpression of hsa_circ_0075542 alleviated the downregulated effect of overexpression of miR-1197 on HOXC11 levels.

### 3.4 Overexpression of miR-1197 played a tumor-promoting role in prostate cancer cells

**Figure 4.**
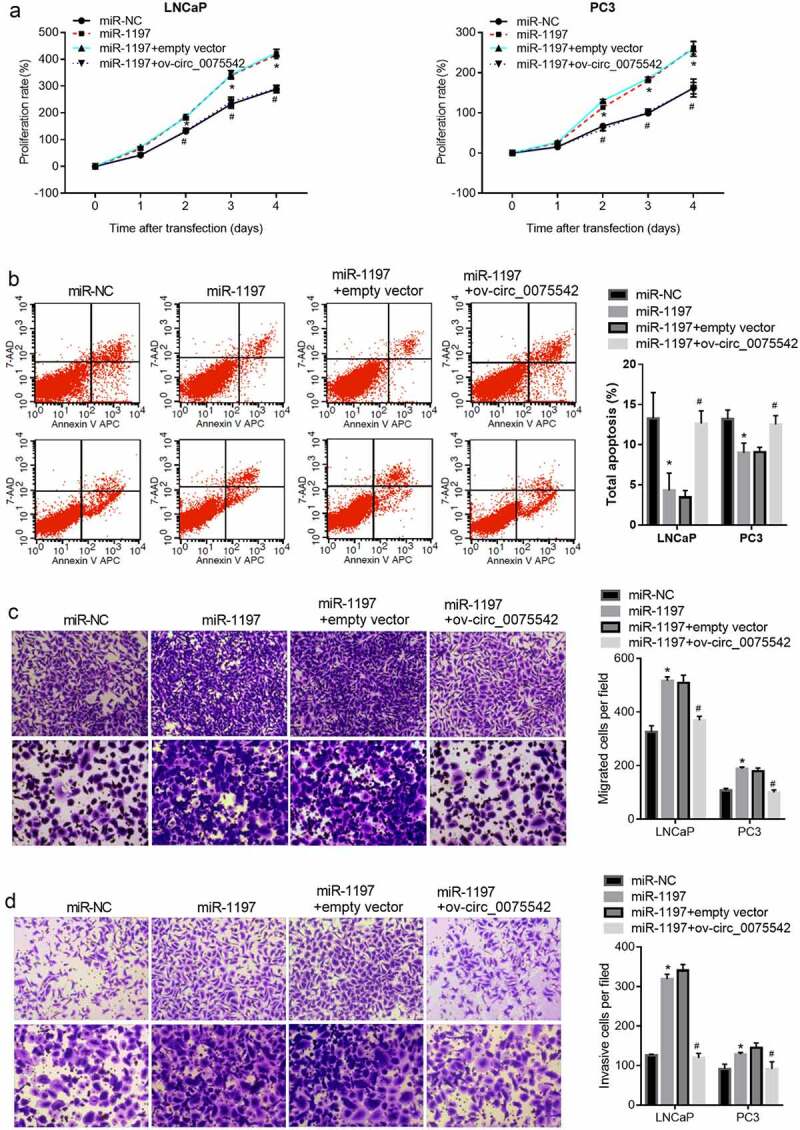
Overexpression of hsa_circ_0075542 alleviated the tumor-promoting effect of miR-1197 overexpression. After being transfected with miR-NC, miR-1197 mimic, miR-1197 mimic plus empty vector, or miR-1197 mimic plus ov-circ_0075542, the effect of this transfection on cell proliferation (a) and apoptosis (b) was assessed using cell counting kit-8 and flow cytometry assays. The capabilities of migration (c) and invasion (d) were evaluated using transwell assays. *P < 0.05, when miR-1197 group vs. the miR-NC group; ^#^P < 0.05, when miR-1197 + ov-circ_0075542 group vs. the miR-1197 + empty vector group

The miR-1197 mimic was transfected into cells to overexpress miR-1197. The proliferation rate of cells transfected with the miR-1197 mimic was higher than that of cells transfected with miR-NC (Figure 4(a)). The apoptotic cell rate of cells transfected with miR-1197 mimic was lower than that of cells transfected with miR-NC (Figure 4(b)). Moreover, the migrated and invasion cell numbers transfected with miR-1197 mimic were higher than those of cells transfected with miR-NC (Figure 4(c)). Therefore, overexpression of miR-1197 promoted malignancy and suppressed apoptosis in prostate cancer cells.

### 3.5 Overexpression of hsa_circ_0075542 alleviated the tumor-promoting effect of miR-1197 overexpression

hsa_circ_0075542 was overexpressed in miR-1197 mimic-transfected cells. The proliferation rate was lower in cells co-transfected with miR-1197 + ov-circ_0075542 than in those co-transfected with miR-1197 + empty vector (Figure 4(a)). Number of apoptotic cells was higher in cells co-transfected with miR-1197 + ov-circ_0075542 than in those co-transfected with miR-1197 + empty vector (Figure 4(b)). Moreover, migrated or invasion cell numbers were lower in cells co-transfected with miR-1197 + ov-circ_0075542 than in those co-transfected with the miR-1197 + empty vector (Figure 4(c)). Therefore, overexpression of hsa_circ_0075542 the tumor-promoting effect of overexpressed miR-1197.

## 4. Discussion

Consistent with the results of a previous study [[14]], the expression of hsa_circ_0075542 was downregulated in prostate cancer tissues, suggesting that hsa_circ_0075542 might mediate the pathological process of prostate cancer. To verify this hypothesis, overexpression of the hsa_circ_0075542 LNCaP and PC3 cell model was simulated. Overexpression of hsa_circ_0075542 suppressed cell proliferation, migration, and invasion and promoted apoptosis. Therefore, hsa_circ_0075542 might act as a tumor suppressor gene in LNCaP and PC3 cells. The function of hsa_circ_0075542 in cancer has not been reported to date. In the present study, hsa_circ_0075542 was first identified as a circRNA and further assessed its function in prostate cancer *in vitro*. Further verifying the role of hsa_circ_0075542 using a mouse model and analyzing its potential role in the diagnosis and prognosis of cancer will be future goals.

Many studies have suggested that circRNA acts as a competitive endogenous RNA of miRNAs; therefore, the regulatory mechanism of hsa_circ_0075542 was explored based on the competitive endogenous RNA mechanism. Functional *in vitro* experiments showed that hsa_circ_0075542 suppressed prostate cancer progression. Therefore, according to the competitive endogenous RNA mechanism, hsa_circ_0075542 might operate through miRNAs that promote cancer progression. Six MREs were found on the hsa_circ_007554 sequence. The downstream miRNAs of hsa_circ_0075542 were initially screened by analyzing the effect of the six miRNAs on PC3 cell proliferation. The results showed that only miR-1197 exerted a promoting effect on cell proliferation. Overexpression of miR-1197 overexpression enhanced the capabilities of migration and invasion and decreased the apoptosis in LNCaP and PC3 cells, indicating tumor-promoting effects in prostate cancer cells. Therefore, hsa_circ_0075542 might play its role by sponging miR-1197.

The results from the luciferase reporter and biotinylated miRNA pull-down assays showed that miR-1197 could bind to hsa_circ_0075542; that is, hsa_circ_0075542 could sponge miR-1197 in LNCaP and PC3 cells. Fluorescence in situ hybridization showed that hsa_circ_0075542 and miR-1197 were co-localized. The overexpression of hsa_circ_0075542 increased the protein levels of HOXC11, which is the direct target of miR-1197 in human non-small cell lung cancer cells [23]. Overexpression of miR-1197 decreased the protein level of HOXC11 (Figure 3(e)), supporting that miR-1197 targeted HOXC11 in prostate cancer cells. Our results showed that overexpression of hsa_circ_0075542 alleviated the tumor-promoting effect of miR-1197 overexpression, suggesting that hsa_circ_0075542 acted as a competitive endogenous RNA of miR-1197 in suppressing prostate cancer progression. Although the other five miRNAs played cancer-promoting roles, hsa_circ_0075542 might be their competitive endogenous RNA.

Based on the above hypothesis, HOXC11 might act as a tumor suppressor in prostate cancer cells. HOXC11 is a member of the homeobox family, which is a highly conserved family of transcription factors [24,25]. It is known that HOXC11 plays a tumor-suppressing role in human non-small cell lung cancer cells [23], whereas it plays a tumor-promoting role in renal clear cell carcinoma and colon adenocarcinoma [26,27]. At present, the role of HOXC11 in prostate cancer cells has not been discussed. The detection of HOXC11 further proves that hsa_circ_0075542 acts as a competitive endogenous RNA of miR-1197. There are many potential downstream genes of miR-1197 that do not act through HOXC11 alone. The downstream target genes of hsa_circ_0075542/miR-1197 signal axis will be identified in future experiments; further, their expression characteristics and functions will be analyzed to further support our conclusions.

## 5. Conclusion

In the present study, expression of hsa_circ_0075542 was downregulated in prostate cancer tissues, and it suppressed malignancy and promoted apoptosis in LNCaP and PC3 cells by acting as a competitive endogenous RNA of miR-1197. The hsa_circ_0075542/miR-1197 axis might play its role via HOXC11. The potential role of hsa_circ_0075542, miR-119 7, and HOXC11 as diagnostic and prognostic markers needs to be analyzed, and their role in needs to be further verified *in vivo* in future research.

## Supplementary Material

Supplemental Material

## Data Availability

All data from this study are available in this published article.

## HIGHLIGHTS

1. hsa_circ_0075542 level is downregulated prostate cancer tissues
2. Over-expression of hsa_circ_0075542 suppresses cell proliferation and metastasis
3. Over-expression of hsa_circ_0075542 promotes apoptosis
4. hsa_circ_0075542 targets the miR-1197/HOXC11 axis

## Abbreviations

circular RNAs (circRNAs); microRNA (miRNA); quantitative reverse transcription PCR (qRT-PCR); overexpression of hsa_circ_0075542 recombinant plasmid (ov-circ_0075542); negative control miRNA (miR-NC); miRNA response element (MRE); biotinylated miR-1197 mimics (biotin-miR-1197;, biotinylated miR-NC mimics (biotin-miR-NC); homeobox C11 (HOXC11)
