# Supplementary material for: Circular RNA hsa_circ_0075542 acts as a sponge for microRNA-1197 to suppress malignant characteristics and promote apoptosis in prostate cancer cells
Source: Bioengineered. 2021 Sep 13;12(1):5620–31. doi: 10.1080/21655979.2021.1967064 (PMC8806842; doi:10.1080/21655979.2021.1967064)
Supplement: Supplemental Material [file KBIE_A_1967064_SM5830.zip › suppl...pdf]

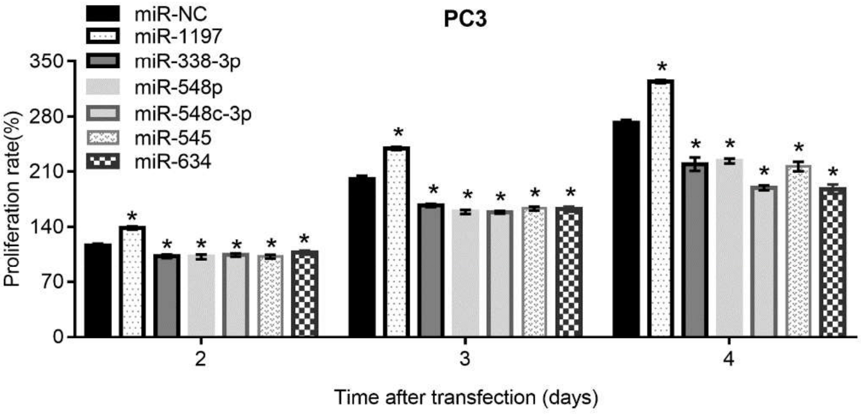

Figure S1. Effect of miR-1197, miR-338-3p, miR-548p, miR-548c-3p, miR-545, and miR-634 on the cell proliferation of PC3 cells. Negative control miRNA (miR-NC), miR-1197 mimic, miR-338-3p mimic, miR-548p mimic, miR-548c-3p mimic, miR-545 mimic, or miR-634 mimic were transfected into PC3 cells. After transfection for 0, 1, 2, 3 and 4 days, cell proliferation was assessed using a cell counting kit-8 assay.

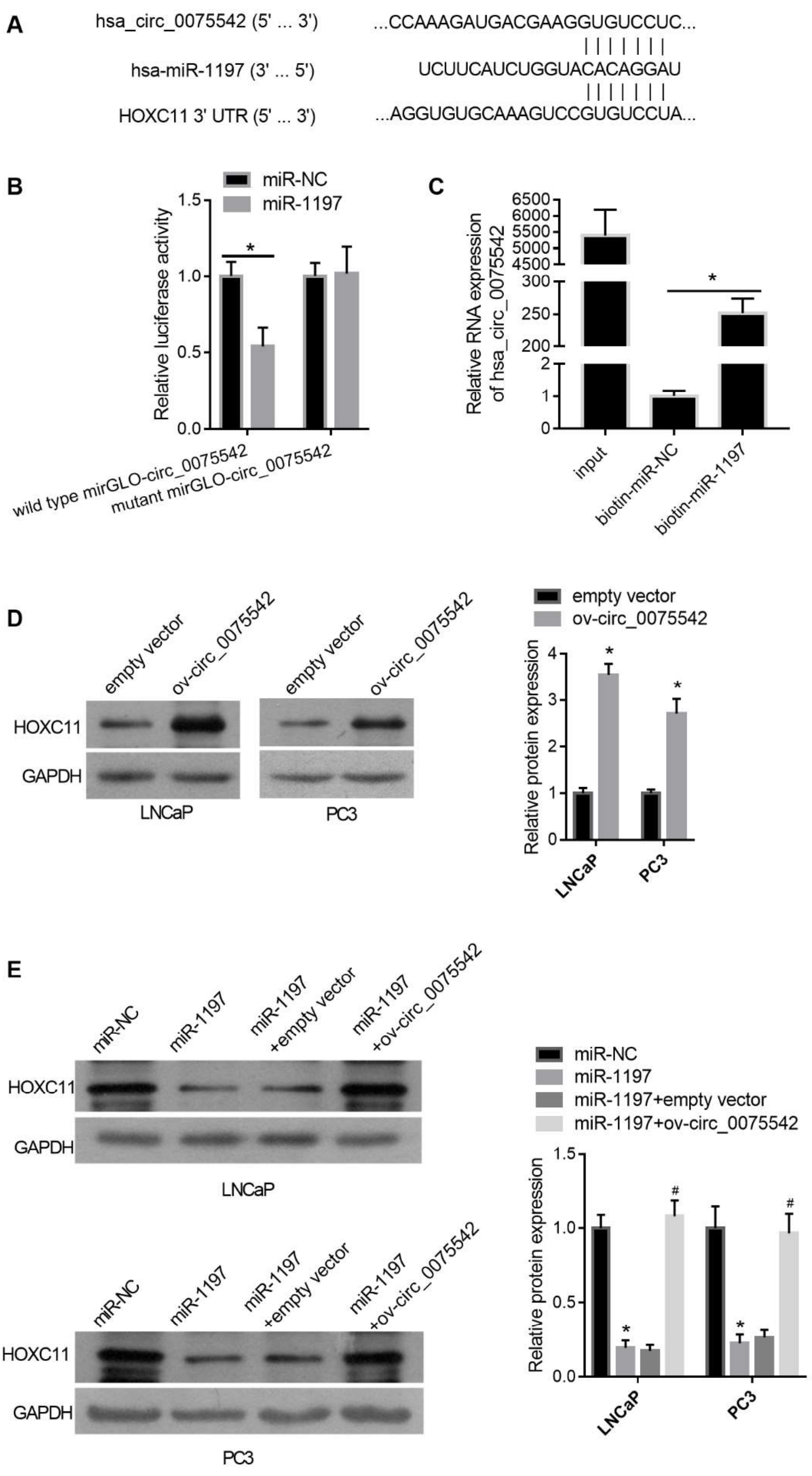
A
hsa_circ_0075542 (5' ... 3')
...CCAAAGAUGACGAAGGUGUCCUC...
hsa-miR-1197 (3' ... 5')
UCUUCAUCUGGUACACAGGAU
HOXC11 3' UTR (5' ... 3')
...AGGUGUGCAAAGUCCGUGUCCUA...
B
miR-NC
miR-1197
Relative luciferase activity
wild type mirGLO-circ_0075542
mutant mirGLO-circ_0075542
C
Relative RNA expression of hsa_circ_0075542
input
biotin-miR-NC
biotin-miR-1197
D
empty vector
ov-circ_0075542
HOXC11
GAPDH
LNCaP
PC3
Relative protein expression
E
miR-NC
miR-1197
miR-1197 +empty vector
miR-1197 +ov-circ_0075542
miR-1197+empty vector
miR-1197+ov-circ_0075542
HOXC11
GAPDH
LNCaP
PC3
Relative protein expression

Figure 3 hsa_circ_0075542 targets the miR-1197/HOXC11 axis. (A): miR-1197 binding sites on hsa_circ_0075542 and 3′-noncoding region of HOXC11 mRNA. (B): Effect of miR-1197 mimic or miR-NC on the luciferase activity of recombinant luciferase reporter. To construct a luciferase reporter, the wild-type linear sequence of hsa_circ_0075542 was cloned into the miRNA target expression vector GP-mirGLO, named wild-type mirGLO-circ_0075542. miR-1197 binding site on wild-type mirGLO-circ_0075542 was mutated and the mutant plasmid was named mutant mirGLO-circ_0075542. miR-NC or miR-1197 was co-transfected with each recombinant luciferase reporter plasmid, and the luciferase activity was measured. (C): Relative expression of hsa_circ_0075542 in pulled-down RNA using biotinylated miR-NC or biotinylated miR-1197 as a probe. The ‘input’ group is the isolated RNA from the partial lysate before the cell pull-down assay. (D): HOXC11 protein level in cells transfected with overexpression of hsa_circ_0075542 vector (ov-circ_0075542) or empty vector pLC5-ciR (empty vector). (E): HOXC11 protein abundance in LNCaP and PC3 cells transfected with miR-NC, miR-1197 mimic, miR-1197 mimic plus empty vector, or miR-1197 mimic plus ov-circ_0075542. *$P < 0.05$.

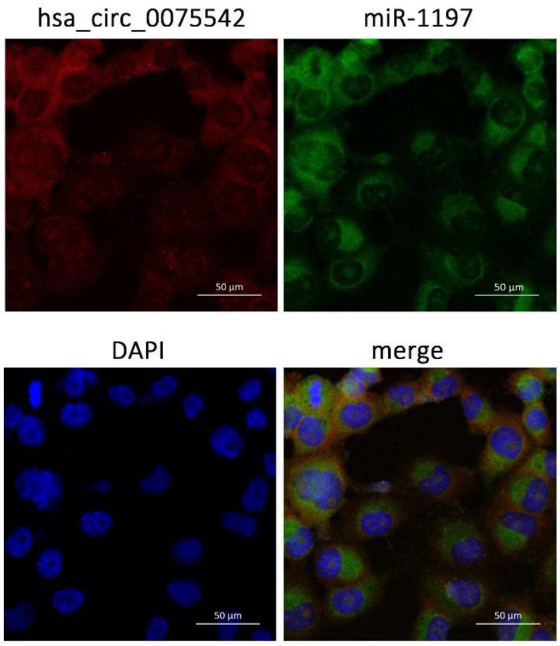

Figure S2. Cellular sublocalization of hsa_circ_0075542 and miR-1197 in PC3 cells. Fluorescence in situ hybridization was performed to analyze cellular sublocalization. Red is the signal of the hsa_circ_0075542

probe labeled with TYE665, green is the signal of the miR-1197 probe labeled with FAM, and blue is the signal of 4’,6-diamidino-2’-phenylindole used to stain the nucleus.

3.4 Overexpression of miR-1197 played a tumor-promoting role in prostate cancer cells

The miR-1197 mimic was transfected into cells to overexpress miR-1197. The proliferation rate of cells transfected with the miR-1197 mimic was higher than that of cells transfected with miR-NC (Fig. 4A). The apoptotic cell rate of cells transfected with miR-1197 mimic was lower than that of cells transfected with miR-NC (Fig. 4B). Moreover, the migrated and invasion cell numbers transfected with miR-1197 mimic were higher than those of cells transfected with miR-NC (Fig. 4C). Therefore, overexpression of miR-1197 promoted malignancy and suppressed apoptosis in prostate cancer cells.
